# Basque Ethnic Identity and Collective Empowerment: Two Key Factors in Well-Being and Community Participation

**DOI:** 10.3389/fpsyg.2020.606316

**Published:** 2020-11-23

**Authors:** Jon Zabala, Susana Conejero, Aitziber Pascual, Itziar Alonso-Arbiol, Alberto Amutio, Barbara Torres-Gomez, Sonia Padoan De Luca, Saioa Telletxea

**Affiliations:** ^1^Department of Basic Psychological Processes and Development, Faculty of Psychology, University of the Basque Country - UPV/EHU, Donostia-San Sebastián, Spain; ^2^Department of Clinical and Health Psychology, and Research Methods, Faculty of Psychology, University of the Basque Country - UPV/EHU, Donostia-San Sebastián, Spain; ^3^Department of Social Psychology, Faculty of Labor Relations and Social Work, University of the Basque Country - UPV/EHU, Leioa, Spain; ^4^Universidad Andres Bello, Faculty of Education and Social Science, Santiago de Chile, Chile; ^5^Departament of Social Psychology, Faculty of Psychology, University of the Basque Country - UPV/EHU, Donostia-San Sebastián, Spain; ^6^Faculty of Labor Relations and Social Work, University of the Basque Country - UPV/EHU, Vitoria-Gasteiz, Spain

**Keywords:** ethnic identity, identity fusion, collective identity, perceived collective efficacy, collective empowerment, personal well-being, social well-being, community participation

## Abstract

Social identity is a factor that is associated with well-being and community participation. Some studies have shown that ethnic identity goes along with empowerment, and that interaction between the two leads to greater indices of well-being and community participation. However, other works suggest a contextual circumstance (i.e., perceiving one’s own group as a minority and/or being discriminated) may condition the nature of these relations. By means of a cross-sectional study, we analyzed the relations of social identification (or identity fusion) and collective psychological empowerment with personal well-being, social well-being and community participation in a sample of Basques. A total of 748 Basques participated (63.1% women; age *M* = 39.28; *SD* = 12.13). Individuals who were highly identified or fused with Basque speakers and who were highly empowered showed higher indices of well-being (both personal and social) and of community participation than non-fused individuals with low empowerment. The results also suggest that social identification (or identity fusion) offsets the negative effects of perceiving the group as a linguistic minority. Collective psychological empowerment proved to be an especially relevant factor that needs to continue to be explored.

## Introduction

Promotion of well-being is clearly a desirable goal for all individuals, and also for all human groups. Unraveling the amalgam of factors that influence well-being is, precisely, one of the great contributions of social sciences (Salvador-Carulla et al., 2014). Among these factors, social identity stands as a remarkable element that has a positive influence on well-being (Haslam et al., 2009; Jetten et al., 2012). Indeed, some studies (e.g., Molix and Bettencourt, 2010; Lardier et al., 2018) have shown that synergy or interaction between ethnic identity and psychological or individual empowerment can contribute to both well-being and to community participation. Notwithstanding, there are other collective factors, such as collective psychological empowerment (CPE) (Drury and Reicher, 2009) that may contribute to well-being and community participation, but that have been less investigated (Staples, 1990; Soares et al., 2015). However, neglecting the role that this sort of social variable plays in the study of well-being may lead to lower awareness of its function and, therefore, lower capacity to develop individuals and societies with greater degrees of well-being. Moreover, understanding how the differential interaction of underlying collective factors affects increased community participation in highly diverse ethnic groups proves essential.

The vast majority of studies that have analyzed social identity and empowerment as related to well-being, have been carried out with the participation of people from vulnerable groups as, for example, African Americans and Latinos in the United States context (Gutiérrez, 1995; Phinney, 1996; Syed and Juang, 2014; Karaś et al., 2015; Lardier, 2018; Lardier et al., 2018). The Basque ethnic group^1^ is a collective which, due to its particular characteristics, sparks great interest for the study of social identity and empowerment. This is a collective that resides in the Basque Country, a region currently split between Spain and France. This group has its own culture and differentiated language (Euskera)^2^, by means of which it has reinforced its identity (Trask, 1997; Roman, 2015). After a great setback and the damage to its culture and language during the Francoist dictatorship (1939–1975), this collective has begun to recover and reinforce its Basque identity to this day (Urla and Burdick, 2018). The countless popular initiatives of Basques are a display of an active collective that is proud of its identity, a collective that has achieved major milestones regarding its culture and language, *Euskera* or Basque (Urla, 2012; Urla and Burdick, 2018), a key aspect in this study.

The Basque collective holds a good socioeconomic position in comparison with other groups in its surroundings (Urla and Burdick, 2018), yet at the same time, we can currently find different indicators of discrimination against and threat to *Euskera*, including prejudice toward the language and a limited use in public life (Gorter et al., 2012). In this work, we refer to Basques as the ensemble of individuals who have a feeling of belonging, closeness and/or affinity toward Basque speakers, *euskaldunak*. Basque speakers whose mother tongue is Euskera account for 15.35% of the Basque Country’s population. However, 29.62% of the inhabitants of the Basque Country have acquired language ability in Euskera, even though it is only practiced by 12.6% in public life (Sociolinguistic Cluster, 2020). This situation makes the Basque community a linguistic minority, whose language (and identity) is in a vulnerable situation and a clear disadvantage compared to Spanish (or French). Despite the prestige that Basque enjoys today (i.e., Euskera has taken on enormous presence in the formal educational sphere and provides clear advantages in accessing numerous job positions, especially in the public sector), this language is perceived by some sectors as an imposed language, difficult to learn and unnecessary on a day-to-day basis, in such a way that for many, learning Basque is based on external and instrumental motivations. Other people reject it because they perceive that it is politicized and, instead of considering it as part of the Basque culture, they associate it with specific political options or ideologies (Amorrortu et al., 2009). The coexistence of both languages, therefore, is not without problems and political tensions. Additionally, another factor to be taken into account in order to understand the nature of the social identity of the Basques is their recent past. Part of the adult population lived through the Francoist dictatorship (1939–1975), and the majority of the people has also lived the period of the armed, social and political crisis of the Basque conflict (1959–2011). So, this is a collective where the linguistic-cultural aspect holds an important central role in its ethnic identity and that, despite that currently it is not suffering from a situation of social stigmatization- as occurs with most ethnic groups analyzed in the literature - (Gutiérrez, 1995; Karaś et al., 2015; Lardier, 2018; Lardier et al., 2018), it is a group of people that has seen the expression of their language violated (Mínguez Alcaide et al., 2014, 2015) and that is currently still working to achieve their full potential.

Taking into account all the above, this study analyzes the relations of social identification (or identity fusion) and CPE with personal well-being, social well-being and community participation in a sample of Basques.

### Social Identity, Social Identification and Identity Fusion, and Their Relation to Well-Being and Community Participation

Firstly, we must make a small yet important distinction between social identity and social identification. *Social identity* customarily defined as “the individual’s knowledge that he [or she] belongs to certain social groups together with some emotional and value significance to him [or her] of the group membership” (Tajfel, 1972, p. 31), is not exactly the same as the *social identification* originally defined by Tajfel (1978) as the positive emotional valuation of the relationship between self and in-group (see Postmes et al., 2013, p. 599). Social identity refers to the group as a (perceived) entity, while social identification is a more specific concept and refers to the relation or subjective affiliation of the individual with the group (Postmes et al., 2005, 2013; Postmes and Jetten, 2006; Leach et al., 2008).

Based on the classic theory of social identity, we understand that personal identity and social identity are antagonistic, meaning that when one of them is activated, the other is eclipsed or canceled (Gómez et al., 2020). This rigid distinction can be fairly problematic (Baray et al., 2009; Swann et al., 2012), since even when speaking of a (hypothetical) purely personal identity, it is difficult for social aspects not to be activated, and vice versa. A person’s identity is inevitably both personal and social (Vignoles, 2011), so an approach that establishes an absolute distinction between both identities, from our perspective, is removed from the psychological and social reality of this phenomenon. In the same fashion, identity fusion theory (Swann et al., 2009) posits that, far from being antagonists, personal and social identity mutually influence each other, so that they can activate and feed back into one another simultaneously. As with social identification, identity fusion theory focuses on the individual and in-group relations, and not on inter-group relations like the classic theory of social identity (Postmes et al., 2013; Gómez et al., 2020). In this sense, we believe that an approach focused on social identification and the theory of identity fusion is necessary.

Identity fusion could be a differentiated construct from social identification (Gómez et al., 2020), but the fact that they are both closely related (Swann et al., 2012; Zumeta et al., 2016; Bortolini et al., 2018), and share aspects with the social identity theory (Leach et al., 2008; Postmes et al., 2013; Gómez et al., 2020) justifies our consideration of identity fusion as part of the processes of social identification and social identity. In Table 1 different concepts are discussed, as well as another series of related terms.

**TABLE 1:** Definitions of “social identity” and other related concepts in relation to personal identity or Self.

References	Denominations	Definitions	Focus on self
	Social identity		
Tajfel (1972)		“(…) the individual’s knowledge that he [or she] belongs to certain social groups together with some emotional and value significance to him [or her] of the group membership” (p. 31).	Low
Thoits and Virshup (1997)		“(…) socially constructed and socially meaningful categories that are accepted by individuals as descriptive of themselves or their group” (p. 106).	High
	Social identification		
Tajfel (1978)		“(…) as the positive emotional valuation of the relationship between self and in group” (p. 28–29).	High
Postmes et al. (2019)		“(…) it is one of the processes by which social identities are internalized” (p. 111).	High
Gómez and Vázquez (2015)	Identity fusion	“(…) a visceral feeling of “oneness” with the group wherein the personal self (characteristics of individuals that make them unique) joins with a social self (characteristics of individuals that align them with a group) and the borders between the two become porous” (p. 482).	High
	Ethnic identity		
Phinney et al. (1996)		“(…) as a complex construct including a commitment and sense of belonging to the group, positive evaluation of the group, interest in knowledge about the group, and involvement in social activities of the group” (p. 168).	High
Phinney (1996)		“(…) fundamental aspect of the self that includes a sense of membership in an ethnic group and the attitudes and feelings associated with that membership” (p. 922).	High
Ashmore et al. (2004)	Collective identity	“(…) is defined here in terms of a subjective claim or acceptance by the person whose identity is at stake (…). That is, although others may refer to one in terms of a particular social category, that category does not become a collective identity unless it is personally acknowledged as self-defining in some respect” (p. 81).	High
Mael and Ashforth (1992)	Organizational identification	“(…) is a specific form of social identification where the individual defines him or herself in terms of their membership in a particular organization” (p. 105).	High
Espinosa and Tapia (2011)	National identification	“(…) national identity is a specific sub-type of social identity” (p. 71).	Low
Cicognani et al. (2015)	Sense of community	“(…) the perception of similarity with others, a recognized interdependence, a willingness to maintain such interdependence offering or making for others what is expected from us, the feeling to belong to a totally stable and reliable structure” (p. 28).	High
Proshansky (1978)	Place identity	“(…) those dimensions of self that define the individual’s personal identity in relation to the physical environment by means of a complex pattern of conscious and unconscious ideas, beliefs, preferences, feelings, values, goals, and behavioral tendencies and skills relevant to this environment” (p. 155).	High

In any event, what is certain is that social identity is an important predictor of a wide range of health and well-being indicators. This is because social identity covers psychological needs that are fundamental for human beings, such as the psychological need for belonging (Greenaway et al., 2016), for social support (Kearns et al., 2017) and for social resources, including shared meanings and ways of understanding (Haslam et al., 2009). Moreover, social identity protects human beings from depression, providing a sense of purpose, meaning and control in life (Dingle et al., 2013; Cruwys et al., 2014; Postmes et al., 2019).

Recent meta-analyses have shown that social identity and/or identification toward different groups (whether ethnic, organizational, national, etc.) are relevant factors for the well-being of people (see Table 2). The evidence suggests that ethnic identity, particularly for those belonging to ethnic minorities is significantly associated with a typical effect of *r* = 0.17 with well-being, regardless of race, gender and socioeconomic status (Smith and Silva, 2011), and of *r* = 0.15 with the absence of depression amongst a population of diverse ethnicity and backgrounds (Postmes et al., 2019). In fact, the data suggest that social identification has a greater impact on psychological well-being than on depression reduction (Smith and Silva, 2011; Steffens et al., 2017). In turn, amongst a population of diverse ethnicity and backgrounds, identification with non-stigmatized collectives protects more from depression than identification with stigmatized collectives (Postmes et al., 2019).

**TABLE 2:** Meta-analysis on relations between social identity (or identification) and well-being.

Authors	Sample	Variables	Studies and sample	Effects size associations	Results
Espinosa et al. (2015)	General population and university students of Argentina, Peru and Mexico.	- National identity.- Social well-being	*K* = 5 studies *N* = 1825	National identification correlates: *r* = 0.21 with social integration *r* = 0.13 with social acceptance *r* = 0.12 with social contribution *r* = 0.16 with social actualization.	Results show evidence of weak but significant relationships between national identification and the different dimensions of social well-being. Effect sizes are heterogeneous between nations.
Postmes et al. (2019)	Population of diverse ethnicity and backgrounds: European, Latino, African-American, European-American, First Nation, various Asian countries and so forth.	- Group identification.- Depression.	*K* = 76 studies *N* = 31,016	Group identification correlates: *r* = −0.15 with less depression *r*_*z*_ = −0.28 with interactive groups *r*_*z*_ = −0.11 with social categories *r*_*z*_ = −0.24 with non-stigmatized groups *r*_*z*_ = −0.10 with stigmatized groups.	Results reveal that group identity has a modest negative association with depression. Studies that focused on identification with interactive groups had larger effect sizes than studies that focused on social categories. Moreover, studies of non-stigmatized groups had larger effect sizes than studies of stigmatized groups.
Smith and Silva (2011)	People of color from USA: 33% African Americans, 35% Asian Americans, 21% Hispanic, 5% Native Americans, 1% Pacific Islander Americans, 5% from “other” non-White groups.	- Ethnic Identity. - Self-esteem. - Coping ability. - Symptoms of depression.	*K* = 184 studies *N* = 41,626	Ethnic identity correlates: *r* = 0.17 with general personal well-being *r* = 0.07 with mental health (distress) *r* = 0.24 with self-esteem *r* = 0.25 with well-being.	Results reveal a modest association between ethnic identity and personal well-being. Ethnic identity correlates more strongly with self-esteem and well-being than with distress. No differences were observed across participant race, gender, or socioeconomic status. These findings support the general relevance of ethnic identity across people of color.
Steffens et al. (2017)	Workers of a large variety of jobs.	- Organizational identification.- Work group identification. - Psychological or physiological health and well-being, including burnout.	*K* = 58 studies *N* = 19,799	Both organizational and work group identification correlate *r* = 0.21 with health *r* = 0.27 with psychological well-being *r* = 0.18 with absence of stress.	Analysis identified a modest relationship between health and for both work group and organizational identification. The relationship is stronger (a) for indicators of the presence of well-being than absence of stress, (b) for psychological than physical health.
Steffens et al. (2019)	People from group interventions enhancing social identification.	- Intervention enhancing social identification. - Well-being and health outcomes.	*K* = 27 studies *N* = 2,230	Social identification-building interventions impact *r* = 0.31 on health *r* = 0.33 increasing self-esteem *r* = 0.32 increasing well-being *r* = −0.23 to *r* = −0.29 reducing stress, anxiety and depression.	Results indicate that social identification-building interventions had a moderate-to-strong impact on health. More impact on increasing physical health and quality of life than reducing stress.

The rejection-identification model suggests that, perceived racial prejudice has negative consequences on the individual but, at the same time, suggests that a strong identification with the own stigmatized group itself can also offset these negative effects and promote well-being (Branscombe et al., 1999). Moreover, a series of studies indicates that ethnic identity can be a more important factor for well-being amongst individuals belonging to discriminated ethnic minorities than for ethnic majorities^3^ or dominant collectives (mainly white people) (Crocker et al., 1994; Phinney, 1996; Carter et al., 2001, 2005; Mossakowski, 2003; Syed and Juang, 2014). In fact, by the simple fact that the group itself (regardless of being discriminated against) is perceived as a minority in-group identification can become more salient, and thus more closely associated to well-being than when the group is majority (Tajfel and Turner, 1986; Phinney et al., 1996). Therefore, according to the literature, it may be that the perception of being a minority and or discriminated also plays a relevant role in ethnic identity (Hipolito-Delgado and Zion, 2017) and its relation to well-being. In this regard, analyzing the existing association between social identification (or identity fusion) and well-being (personal and social) in a linguistic minority, but not necessarily a stigmatized group, takes on special value.

Lastly, social identification is also an important factor associated with greater commitment, participation and pro-group behaviors (Chavis and Wandersman, 2002; Perkins et al., 2002; Shamir and Kark, 2004; Gómez et al., 2011; Swann et al., 2012; Speer et al., 2013; Christens and Lin, 2014). A meta-analysis (Talò, 2018) concluded that three of the six factors most associated with community participation were aspects related to social identification (Vignoles, 2011): sense of community, community identity and place identity.

### Psychological Empowerment (PE) and Collective Psychological Empowerment (CPE)

Gutiérrez (1995) defines psychological empowerment (PE) as “the process of increasing personal, interpersonal, or political power, so that individuals, families and communities can take action to improve their situations” (p. 229). Most studies that fall under the social nature of empowerment have conceptualized and analyzed PE in individual terms or from the perspective of the power an individual perceives they hold through social structures (organizations and communities) (Perkins and Zimmerman, 1995; Molix and Bettencourt, 2010; Christens et al., 2011; Lardier et al., 2018). However, empowerment must also be analyzed and conceptualized in collective terms (Staples, 1990). In fact, the main components of PE are group identification, group consciousness and perceived collective efficacy (Gutiérrez, 1995). Moreover, change and social justice are viewed as a means of empowerment and achieved through collective work (Gutiérrez, 1995; Speer and Hughey, 1995; van Zomeren et al., 2008; Drury and Reicher, 2009; Christens et al., 2011; Peterson, 2011).

Drury and Reicher (2009) define CPE as a positive social psychological transformation related to a sense of being able to shape the social world. This transformation occurs for members of subordinated groups who override existing relations of dominance. Group identification is the driving force behind CPE, and empowerment is experienced as a life-changing experience. Thus, through this experience, individuals perceive themselves as more confident and empowered at an individual level, as well. A concept similar to CPE is Bandura’s (1997) perceived collective efficacy. Perceived collective efficacy is defined as a shared belief of a group in its ability to organize and carry out the actions required to reach certain objectives. With CPE, the shared belief of group members would be at least partially based on the conviction that a social change can be made and that political power can be increased (Drury and Reicher, 2009).

### Relation of Perceived Collective Efficacy, Empowerment and Social Identification With Well-Being and Community Participation

Prior studies have shown that both *self-efficacy* and *perceived collective efficacy* may be related, and that both are associated with greater psychological well-being, self-esteem, life satisfaction, better stress management (Bandura, 1997; Devonport and Lane, 2006; Siu et al., 2007; Viel-Ruma et al., 2010; Çalik et al., 2012; Roos et al., 2013) and intense positive emotions (Zumeta et al., 2020). In turn, the study by Soares et al. (2015) showed that individual empowerment was also related to collective empowerment, and that both of them were associated with higher quality of life and better stress management. However, while individual empowerment was only associated with the psychological aspect of quality of life (e.g., self-esteem, body image and physical appearance, feelings), collective empowerment was associated with all aspects of quality of life, both in psychological, as well as physical, social and environment terms.

Other studies have shown that social identification, PE, collective efficacy, well-being and community participation are factors that are closely related (Ohmer, 2007; Cicognani et al., 2008, 2015; Tamanas, 2010; Christens et al., 2011; Speer et al., 2013; Christens and Lin, 2014). Additionally, in the field of organizations, other studies have shown that the relations between organizational identification and well-being at work are mediated by perceived collective efficacy (Borgogni et al., 2010; van Dick and Haslam, 2012; Avanzi et al., 2015).

Lastly, the work by Lardier et al. (2018) showed that a strong ethnic identity in African Americans was associated with higher PE (specifically, greater sociopolitical control). Additionally, participants with high scores in ethnic identity and also in PE had a lower risk of drug consumption, higher community participation and a greater sense of community than participants with lower scores in ethnic identity and PE. Moreover, through a structural equations model, Lardier (2018) showed that ethnic identity was a mediator for the relation existing between the sense of community and community participation with PE.

In the same line as the study by Lardier et al. (2018) and Molix and Bettencourt (2010) found that relations between ethnic identity, PE and well-being were different between ethnic minorities in the United States and white Americans. While higher levels of ethnic identity predicted greater PE and greater well-being amongst ethnic minorities, these relations were scant and unreliable amongst white participants. Moreover, for ethnic minorities, the relation between ethnic identity and well-being was significantly mediated by PE, which was not the case for the white American group.

As a whole, these results suggest that the influence of different forms of social identification on well-being and community participation may be the result, at least partially, of the association between social identification and empowerment. Furthermore, the studies by Molix and Bettencourt (2010) showed that these relations may be stronger in minority collectives than in dominating majority collectives.

## Objectives and Hypotheses

The main objective of this study was to analyze the relationship between identification with Basque speakers or ethnic identity fusion (EIF), as well as CPE with personal well-being, social well-being and community participation in a group of Basques. It was predicted that: i) EIF and CPE will be closely related (H1); ii) EIF and CPE will have a positive relationship with the following variables: personal well-being (H2a), social well-being (H2b) and community participation (H2c); iii) Both EIF and CPE will have a positive synergistic or interactive effect on well-being (personal and social) (H3a) and on community participation (H3b), such that the participants fused with Basque speakers who are highly empowered will have higher indices of well-being and community participation than the rest of participants. In turn, in an exploratory fashion, we will analyze the effect of perception of integration of the Basque Language –*Euskera*- in the socio-cultural environment, as a measurement of the group’s perception as a linguistic minority or majority. Firstly, we will explore correlations with different variables separately (in the group perceiving themselves as a minority versus those who perceive themselves as a majority). Furthermore, the possible synergistic or interactive effect of the perception of the linguistic minority-majority and of the EIF on well-being and community participation will be analyzed. Regarding this objective, based on the rejection-identification model (Branscombe et al., 1999) and the meta-analytical results (Smith and Silva, 2011), no hypothesis is set forth because there is evidence in both directions: identification may be greater and more positive when one forms part of a minority, and this could be a factor more closely associated with well-being and community participation, or the opposite effect may occur. Moreover, the aforementioned specific linguistic context of the present study (linguistic minority ethnic group, but not suffering socioeconomic or employment discrimination) represents a particularity that, to our knowledge, has not been previously analyzed.

## Materials and Methods

### Participants

This cross-sectional study is part of another broader work of research on the *Korrika*, a mass recreational, non-competitive race that runs through the Basque Country every two years to defend and promote the Basque language. During the race, individuals and/or representatives from different popular associations or institutions pass a baton, so that this “race” shares the message conveying that Euskera never stops, thanks to the collaboration of all those participating. A total of 748 people (63.8% women and 36.2% men) participated in this study, with ages ranging between 18 and 73 years (*M* = 39.28; *SD* = 12.13), who intended to take part in the aforementioned event. Moreover, all participants were residents in the Basque Country.

### Procedure

*Alfabetatze eta Euskalduntze Koordinakundea* (AEK; the association that organizes the *Korrika* event) collaborated with the research team by sending an email invitation to all *Korrika* collaborators, requesting their voluntary participation in the research. On the other hand, researchers went to the capitals of the Basque Country and collected the emails from those who had been willing to participate in *Korrika* and in this research. Participants responded to the survey online through the Qualtrix XM platform three weeks before the *Korrika* event began.

### Measures

All instruments were translated from Spanish to Euskera with the reverse translation method (Balluerka et al., 2007). First, the Spanish scales were translated into Basque independently by two authors with a broad command of Spanish and Basque, and who were familiar with both cultures. Subsequently, the two translations were compared and discussed until a consensual Basque version of the scales was reached. Second, two other authors independently translated all the scales of the Basque version into Spanish (reversed method) and reached a consensual version of it. Finally, the four team members, with the supervision of a Basque philologist, compared each of the scales (original and inversely adapted), ensuring that the meaning of the items was the same.

The scales in Basque were tested with a pilot study with a Basque-speaking sample. Alternatively, participants in the present study had the option of completing the survey in Spanish, although most did so in Euskera (*n* = 630). As seen in Table 3, the reliability of the scales ranged from α = 0.73 to α = 0.88.

**TABLE 3:** Matrix of correlations, descriptive statistics and reliability of scales.

	1	2	3	4	5	6	7	*7.1*	*7.2*	*7.3*	*7.4*	8
1. Age	−	0.07	0.07	−0.18**	−0.08*	−0.10**	0.003	−0.05	0.07	0.07	0.06	−0.20**
2. EIF CA		−	0.86**	0.13**	0.12**	0.11**	0.09**	0.14**	0.10**	0.05	−0.04	0.17**
3. EIF CON			−	0.13**	0.12**	0.15**	0.13**	0.19**	0.11**	0.10**	−0.005	0.16**
4. CPE				−	0.08*	0.47**	0.35**	0.29**	0.26**	0.21**	0.20**	0.19**
5. LMP					−	0.14**	0.10**	0.16**	0.03	0.05	0.04	0.21**
6. Personal well-being						−	0.41**	0.34**	0.26**	0.34**	0.19**	0.18**
7. Social well-being							−	0.71**	0.68**	0.64**	0.69**	0.31**
*7.1. Social integration*								−	0.35**	0.44**	0.23**	0.45**
*7.2. Social acceptance*									−	0.16**	0.39**	0.06
*7.3. Social contribution*										−	0.14**	0.39**
*7.4. Social actualization*											−	−0.007
8. Community participation												−
*Asymmetry*	0.39	0.14	−0.85	−0.52	0.46	−0.56	0.05	−0.44	−0.21	−0.21	0.11	−0.32
*Kurtosis*	−0.57	−1.98	0.18	0.72	−0.56	0.30	0.08	0.25	0.24	−0.06	−0.23	−0.41
*Average*	39.16	1.46	4.29	7.66	2.81	7.77	3.29	3.9	3.14	3.55	2.52	3.59
*SD*	12.12	0.50	0.78	1.27	1.08	1.10	0.46	0.60	0.63	0.70	0.78	0.81
*Cronbach*α	−		−	0.88	−	0.88	0.78	0.68	0.56	0.77	0.83	0.73

*N* = 742. EIF CA, Ethnic Identity Fusion as a categorical variable (fused-non-fused); EFI CON, Ethnic Fusion Identity as a continuous variable; CPE, Collective Psychological Empowerment; LMP, Linguistic Minority-Majority Perception; *SD*, Standard Deviation. *p < 0.05 and **p < 0.01.

#### Ethnic Identification-Ethnic Identity Fusion

The Identity Fusion (Swann et al., 2009) pictographic measure was used. This instrument contains five images. Each one of them has two circles representing the degree of fusion between the Self and the Group, with different degrees of overlapping between both circles. The response range goes from 1 to 5, such that a higher level of identity fusion (overlapping between circles) matches a higher score. Participants selected the image that best represented the relationship they felt toward Basque-speaking individuals, *euskaldunak*. This measure was used as a continuous measurement, which indicated the degree of identification with Basque speakers. Moreover, participants who were totally fused with the group (those who chose the fifth drawing) were categorically differentiated from participants who selected the other categories, in line with the identity fusion theory. This theory posits that there is a qualitative leap between fused and non-fused individuals, such that fused individuals are the ones who would differentiate from others in feeling a strong feeling of collective Basque identity.

#### Collective Psychological Empowerment-Perceived Collective Efficacy

The study based on the Collective Efficacy Questionnaire for Sports (CEQS, Martínez et al., 2011) was adapted to the context and used. Participants followed these instructions: “State to which extent you believe *euskaldunak* are capable of the following things” on a 10-point scale (1 = *Not capable at all* and 10 = *Very capable*). The items were the following: “Being together,” “Carrying out actions,” “Having a positive attitude,” and “Showing enthusiasm.” Moreover, two items made *ad hoc* were added, which attempt to address the cognitive aspect of CPE as understood by Drury and Reicher (2009) on the perception of the collective’s ability to transform society and achieve the collective’s objectives: “Fomenting social change” and “Achieving shared objectives.”

### Perception of Linguistic Minority-Majority

Participants were asked to rate how Basque-speaking or *euskaldun* they thought the community (town or neighborhood) they lived in was on a 5-point scale: 1 = *Not euskaldun or almost not euskaldun*, 2 = *A little euskaldun*, 3 = *Fairly euskaldun*, 4 = *Very euskaldun* or 5 = *Totally euskaldun*). This measure was designed *ad hoc*.

#### Personal Well-Being

The Pemperton Happiness Index (Hervás and Vázquez, 2013) was used to assess two dimensions of well-being: *General well-being* and *Eudaimonic well-being*. We used a 10-point response scale (1 = *Totally disagree* and 10 = *Totally agree*), (e.g., “I think my life is useful and worthwhile”; “I feel very connected to the people around me”).

#### Social Well-Being

The brief Social Well-Being scale was used (Keyes, 1998; adapted by Blanco and Díaz, 2005). Four of the five dimensions of the original scale were measured: Social Integration (a feeling of belonging and being accepted – e.g., “I feel I belong to something I could call a community – neighborhood, town or the Basque Country)”; Social Acceptance (an accepting view of human nature and trust in others – e.g., “I believe that people are kind”); Social Contribution (a belief in having something to contribute to society – e.g., “I have something valuable to give to the world”); Social Actualization (a belief in the potential and growth of society, and therefore a feeling of hope about its future – e.g., “The world is becoming a better place for everyone”). The response scale ranged from 1 = *Not at all* to 5 = *A lot*. In the Euskera and Spanish version of this scale, the items worded in negative terms were inverted to improve comprehension (García and Magaz, 2009).

#### Community Participation

In order to measure the habit of participating in the community and in collective Basque events, the following three items were created *ad hoc*: “I collaborate in some way in certain initiatives (festivals, events, special days, celebrations, races, etc.) in my town or neighborhood, organizing, working, having meetings, etc.,” “I attend or take part in some way in certain initiatives (festivals, initiatives, special days, celebrations, races…) in my town or neighborhood” and “I participate in mass initiatives (demonstrations, initiatives, races, celebrations, competitive activities: *Basque pelota* soccer games, *bertsolaritza*, etc.) that are related to the Basque Country.” We used a 5-point response scale (1 = *Never or rarely*, 2 = *Every so often*, 3 = *Sometimes*, 4 = *Often*, 5 = *Always or almost always*).

### Data Analysis

#### Language and Gender Differences

Before proceeding to the main analyses, we verified the possible effect of the language used to complete the questionnaires (Spanish vs Euskera) and gender in correlations between variables examined in this study. Differences in correlations between participants (Spanish vs Euskera) were not statistically significant. In the case of gender, only one significant difference was observed in the association between perception of linguistic minority-majority and social acceptance (*r* = 0.18 for men and *r* = −0.05 for women, *z* = 3.01; *p* = 0.002). As such, subsequent analyses were conducted, considering the sample as a whole. All analyses were carried out with SPSS 25.0.

#### Group Profile Creation

Regarding EIF, and following the recommendations of Swann et al. (2009), participants who felt complete fusion between the self and Basque speakers were assigned to participant group “Fused.” All other participants who felt lesser fusion between the self and Basque speakers were assigned to the participant group “Not Fused.” Additionally, regarding CPE, two groups were created: a group with high scores “Highly Empowered” (40% of the participants with the highest scores), and a group with low scores “Not Very Empowered” (40% of the lowest scores). Thus, we eliminated 20% of the participants with intermediate scores from the analysis, in order to achieve a more refined analysis. Finally, we created another variable by combining “Fused vs Not Fused” and “Highly Empowered vs Not Very Empowered,” which gave way to the following four categories: Fused and Highly Empowered (Group 1); Not Fused and Highly Empowered (Group 2); Fused and Not Very Empowered (Group 3) and, lastly, Not Fused and Not Very Empowered (Group 4). In turn, each group was assigned a score based on the third hypothesis to contrast the interaction (Rosenthal and Rosnow, 2008): Group 1 = 4; Group 2 = −1; Group 3 = −1; Group 4 = −2.

On the other hand, two groups were created based on the perception of linguistic minority-majority: participants who ranked 1 (*Not at all or almost not euskaldun*) or 2 (*Slightly euskaldun*) were assigned to the participant group “Perceived Minority,” and those who ranked 4 (*Euskaldun*) or 5 (*Very euskaldun*) were assigned to the participant group “Perceived Majority.” Participants who ranked 3 (*Fairly euskaldun*) were eliminated from the analyses, since these were participants with intermediate scores.

Another variable was created by combining the perception of linguistic minority-majority and fused-not fused, bearing the following four categories: Perceived Minority and Fused (Group 1); Perceived Minority and Not Fused (Group 2); Perceived Majority and Fused (Group 3), and, lastly, Perceived Majority and Not Fused (Group 4). Additionally, the following scores were assigned, based on the rejection-identification model (Branscombe et al., 1999), as counterweights for interaction (Rosenthal and Rosnow, 2008): Group 1 = −1; Group 2 = −2; Group 3 = 4; Group 4 = −1.

Lastly, descriptive analyses and correlations between the different variables in the entire sample were performed and a MANOVA was conducted to analyze the effect and interaction of the EIF (Fused-Not Fused) and the CPE (Highly Empowered-Not Very Empowered) on the criterion variables. The same correlations were performed separately for each group (Perceived Minority vs Perceived Majority), and another MANOVA was conducted to analyze the effect and interaction of the perception of linguistic minority-majority (Perceived Minority and Perceived Majority) and EIF (fused/non-fused).

## Results

### Correlations Between Variables in the Entire Sample

Firstly, the correlations between the different variables in the entire sample were analyzed (see Table 3). The EIF (as a continuous variable) and CPE had an average-low positive association (H1). Moreover, the EIF had average-low positive correlations with the other criterion variables (except for the social well-being dimension: social actualization) (H2a, H2b, and H2c). As far as the CPE is concerned, it resulted in stronger positive associations with all the aforementioned variables. Of note are CPE’s correlations with personal well-being (H2a) and social well-being (H2b), which were high, especially for personal well-being (*r* = 0.47, *p* < 0.001), and a bit more modest regarding community participation (H3b). These results offer support to the first two hypotheses. In addition, the perception of linguistic minority-majority also resulted in positive associations with EIF, with CPE, and with other criterion variables, except for three dimensions of social well-being (social acceptance, social contribution, and social actualization). Lastly, it is interesting to note that the EIF as a categorical variable (fused-not fused) generally showed correlations that were slightly lower when compared with the EIF as a continuous variable.

### Effect of EIF and EPC on Criterion Variables

The EIFs effect was significant, Wilks’ lambda = 0.943, *F*(6, 549) = 5.515, *p* < 0.001,$\eta_{p}^{2}$ = 0.057, as well as the CPE’s, Wilks’ lambda = 0.788, *F*(6, 549) = 24.545, *p* < 0.001,$\eta_{p}^{2}$ = 0.212, although the size of the effect with EIF was small. The interaction between these variables was not statistically significant, Wilks’ lambda = 0.99, *F*(6, 549) = 0.926, *p* < 0.476,$\eta_{p}^{2}$ = 0.01. However, when applying the aforementioned contrast (Group 1 = 4; Group 2 = −1; Group 3 = −1; Group 4 = −2), significant effects of the *r* were found on criterion variables (except for the social well-being dimension: social actualization), which suggests that there may be some interaction between EIF and CPE (see Table 4). Moreover, to test the third hypothesis, *post hoc* analyses were conducted using the independent variable coming from the combination of the “Fused-Not Fused” profile and the “Highly Empowered-Not Very Empowered” profile (four groups or categories). *Post hoc* comparisons showed that participants from Group 1 (Fused and Highly Empowered) had significantly higher scores in all criterion variables than Group 3 (Fused and Not Very Empowered), and also than Group 4 (Not Fused and Not Very Empowered), but in this latter case, the social actualization dimension was excepted. Likewise, although Group 1 (Fused and Highly Empowered) scored higher than Group 2 (Not Fused and Highly Empowered) on almost all variables, the difference was only statistically significant in the social integration and community participation dimensions.

**TABLE 4:** Post hoc comparisons between EIF and CPE-profile groups in criterion variables.

Variables	Group 1: Fused and Highly Empowered (*n* = 167)	Group 2: Not Fused and Highly Empowered (*n* = 143)	Group 3: Fused and Not Very Empowered (*n* = 106)	Group 4: Not Fused and Not Very Empowered (*n* = 142)	*r*	Differences in the Average	$\eta_{p}^{2}$
Personal well-being	*M* = 8.30 (*SD* = 1.00)	*M* = 8.20 (*SD* = 0.90)	*M* = 7.29 (*SD* = 1.04)	*M* = 7.18 (*SD* = 1.12)	0.32**	1, 2 > 3***, 4***	0.20
Social well-being	*M* = 3.43 (*SD* = 0.49)	*M* = 3.40 (*SD* = 0.43)	*M* = 3.18 (*SD* = 0.33)	*M* = 3.12 (*SD* = 0.42)	0.23**	1, 2 > 3***,4***	0.09
SW: *Social integration*	*M* = 4.14 (*SD* = 0.57)	*M* = 4.01 (*SD* = 0.60)	*M* = 3.90 (*SD* = 0.51)	*M* = 3.70 (*SD* = 0.59)	0.24**	1 > 2*, 3**, 4*** 2 > 4*** 3 > 4**	0.08
SW: *Social acceptance*	*M* = 3.28 (*SD* = 0.62)	*M* = 3.24 (*SD* = 0.67)	*M* = 3.08 (*SD* = 0.58)	*M* = 2.90 (*SD* = 0.62)	0.18**	1 > 3**, 4*** 2 > 3*, 4*** 3 > 4*	0.06
SW: *Social contribution*	*M* = 3.72 (*SD* = 0.77)	*M* = 3.68 (*SD* = 0.62)	*M* = 3.50 (*SD* = 0.67)	*M* = 3.38 (*SD* = 0.64)	0.14**	1 > 3*, 4*** 2 > 4***	0.04
SW: *Social actualization*	*M* = 2.60 (*SD* = 0.83)	*M* = 2.66 (*SD* = 0.81)	*M* = 2.24 (*SD* = 0.67)	*M* = 2.48 (*SD* = 0.73)	0.07	1 > 3*** 2 > 3***, 4* 4 > 3*	0.04
Community participation	*M* = 3.84 (*SD* = 0.74)	*M* = 3.58 (*SD* = 0.87)	*M* = 3.64 (*SD* = 0.79)	*M* = 3.35 (*SD* = 0.85)	0.20**	1 > 2**, 3*, 4*** 3 > 4**	0.05

SD, Standard deviation; SW, Social well-being. Wilks’ Lambda, 0.73, *F* (18, 1553.292) = 10.072, *p* < 0.001,$\eta_{p}^{2}$ = 0.099. *p < 0.05, **p < 0.01, and ***p < 0.001.

### Effects of Linguistic Minority-Majority Perception and EIF on Criterion Variables

With the sub-sample perceived as a linguistic minority, associations of EIF with personal well-being and social integration remained statistically significant. However, the association with other variables lost statistical significance. On the other hand, with the sub-sample perceived as a linguistic majority, associations of EIF with CPE, with social well-being, with the social acceptance dimension and with community participation remained statistically significant, but ceased to be statistically significant with respect to the other variables (personal well-being, social integration and social contribution dimensions) (see Tables 5, 6). Neither in the general sample nor in any of the sub-samples was the relationship between EIF and the social actualization dimension significant. On the other hand, relations between CPE and other variables persisted in both sub-samples, except for EIF in the sub-sample that perceived itself as a minority, as mentioned. Despite all the differences between both sub-samples at a descriptive level, these differences were not statistically significant (e.g., *z* = 1.11; *p* = 0.263 for the relation between EIF and CPE).

**TABLE 5:** Matrix of correlations, descriptive statistics and reliability of the scales with the sub-sample that perceives itself as a Linguistic Minority in its environment.

	EIF CON	CPE	PW	SW	*IN*	*AC*	*CON*	*ACT*	CP
EIF CON	−	0.08	0.13*	0.06	0.16**	0.05	0.04	−0.06	0.09
EPC		−	0.44**	0.34**	0.27**	0.25**	0.25**	0.15**	0.18**
*Asymmetry*	−0.82	−0.57	−0.52	−0.19	−0.46	−0.32	−0.41	0.03	−0.27
*Kurtosis*	0.33	0.99	0.14	−0.09	0.31	0.29	0.27	−0.26	−0.53
*Average*	4.21	7.52	7.58	3.24	3.86	3.12	3.52	2.48	3.42
*SD*	0.80	1.31	1.14	0.45	0.60	0.61	0.69	0.76	0.82
*Cronbach*α	−	0.89	0.89	0.76	0.64	0.52	0.74	0.83	0.70

*N* = 342. EIF CON, Ethnic Identity Fusion as continuous variable; CPE, Collective Psychological Empowerment; PW, Personal Well-being; SW, Social Well-Being; *IN*, Social Integration; *AC*, Social Acceptance; *CON*, Social Contribution; *ACT*, Social Actualization; CP, Community Participation; *SD*, Standard Deviation. *p < 0.05 and **p < 0.01.

**TABLE 6:** Matrix of correlations, descriptive statistics and reliability of the scales with the sub-sample that perceives itself as a Linguistic Majority in its environment.

	EIF CON	CPE	PW	SW	*IN*	*AC*	*CON*	*ACT*	CP
EIF CON	−	0.18**	0.14	0.15*	0.08	0.16*	0.10	0.04	0.17*
EPC		−	0.52**	0.34**	0.31**	0.20**	0.26**	0.15*	0.31**
*Asymmetry*	−1.10	−0.11	−0.61	0.28	−0.38	−0.42	−0.06	0.16	−0.44
*Kurtosis*	0.59	−0.27	0.59	0.08	−0.09	0.31	−0.28	−0.14	−0.20
*Average*	4.42	7.76	7.91	3.33	4.07	3.16	3.60	2.5	3.80
*SD*	0.74	1.13	1.01	0.45	0.59	0.66	0.69	0.79	0.78
*Cronbach*α	−	0.85	0.87	0.77	0.69	0.60	0.77	0.85	0.78

*N* = 190. EIF CON, Ethnic Identity Fusion as continuous variable; CPE, Collective Psychological Empowerment; PW, Personal Well-being; SW, Social Well-Being; *IN*, Social Integration; *AC*, Social Acceptance; *CON*, Social Contribution; *ACT*, Social Actualization; CP, Community Participation; *SD*, Standard Deviation. *p < 0.05 and **p < 0.01.

In addition, MANOVA shows that the effect of the EIF variable (Fused vs Not Fused) was significant (Wilks lambda = 0.96, *F*(7, 504) = 2.993, *p* < 0.004, $\eta_{p}^{2}$ = 0.04), as well as the effect of the perceived linguistic minority-majority (Wilks’ lambda = 0.944, *F*(7, 504) = 4.294, *p* < 0.001, $\eta_{p}^{2}$ = 0.06). The interaction between both variables was not significant = 0.99, *F*(7, 504) = 0.513, *p* < 0.825, $\eta_{p}^{2}$ = 0.007). However, upon applying the aforementioned contrast (Group 1 = −1; Group 2 = −2; Group 3 = 4; Group 4 = −1), significant effects of the *r* on the criterion variables were found, excepting social contribution and social actualization dimensions, which suggests that there could be some interactions between EIF and perceived linguistic minority-majority on the other variables (see Table 7).

**TABLE 7:** Post hoc comparisons between EIF and Linguistic Minority-Majority Perception profile groups in criterion variables.

Variables	Group 1: Minority and Fused (*n* = 138)	Group 2: Minority and Not Fused (*n* = 192)	Group 3: Majority and Fused (*n* = 102)	Group 4: Majority and Not Fused (*n* = 82)	*r*	Differences in averages		$\eta_{p}^{2}$
CPE	*M* = 7.70 (*SD* = 1.21)	*M* = 7.43 (*SD* = 1.36)	*M* = 7.89 (*SD* = 1.17)	*M* = 7.55 (*SD* = 1.05)	0.13 **	1 > 2*1 = 3 = 42 = 4	3 > 2**	0.02
Personal well-being	*M* = 7.74 (*SD* = 1.11)	*M* = 7.49 (*SD* = 1.15)	*M* = 8.01 (*SD* = 1.00)	*M* = 7.80 (*SD* = 1.00)	15**	1 > 2*1 = 3 = 42 < 4*	3 > 2***	0.03
Social well-being	*M* = 3.27 (*SD* = 0.43)	*M* = 3.23 (*SD* = 0.43)	*M* = 3.36 (*SD* = 0.47)	*M* = 3.29 (*SD* = 0.43)	0.09*	1 = 2 1 = 3 = 42 = 4	3 > 2*	0.01
*SW: Social integration*	*M* = 3.96 (*SD* = 0.51)	*M* = 3.80 (*SD* = 0.62)	*M* = 4.08 (*SD* = 0.62)	*M* = 4.03 (*SD* = 0.55)	0.14**	1 > 2**1 = 3 = 42 < 4**	3 > 2***	0.03
*SW: Social acceptance*	*M* = 3.17 (*SD* = 0.60)	*M* = 3.07 (*SD* = 0.62)	*M* = 3.24 (*SD* = 0.62)	*M* = 3.06 (*SD* = 0.71)	0.09*	1 = 2 = 3 = 4	3 > 2 *	0.01
*SW: Social contribution*	*M* = 3.54 (*SD* = 0.69)	*M* = 3.52 (*SD* = 0.66)	*M* = 3.62 (*SD* = 0.75)	*M* = 3.58 (*SD* = 0.60)	0.03	1 = 2 = 3 = 4		0.003
*SW: Social actualization*	*M* = 2.41 (*SD* = 0.73)	*M* = 2.55 (*SD* = 0.76)	*M* = 2.49 (*SD* = 0.80)	*M* = 2.50 (*SD* = 0.79)	-0.002	1 = 2 = 3 = 4		0.005
Community participation	*M* = 3.55 (*SD* = 0.76)	*M* = 3.34 (*SD* = 0.85)	*M* = 3.86 (*SD* = 0.75)	*M* = 3.70 (*SD* = 0.81)	0.20**	1 > 2*3 = 41 < 3**1 = 4 2 < 4***	3 > 2***	0.06

CPE, Collective Psychological Empowerment; SW, Social Well-Being; EIF, Ethnic Identity Fusion. Wilks’ Lambda = 0.887, *F* (21, 1447.766) = 2.953, *p* < 0.001,$\eta_{p}^{2}$ = 0.039. *p < 0.05, **p < 0.01, and ***p < 0.001.

Lastly, a *post hoc* analysis was conducted, using the combined variable of linguistic minority-majority and fused-not fused to this end. On the one hand, Group 1 (Perceived Minority and Fused) scored higher than Group 2 (Perceived Minority and Not Fused) on the CPE, on personal well-being, on the social integration dimension and on community participation. On the other hand, Group 3 (Perceived Majority and Fused) showed no significant difference in any of the variables in comparison with Group 4 (Perceived Majority and Not Fused). In turn, Group 1 (Perceived Minority and Fused) also did not differ in comparison with Group 3 (Perceived Majority and Fused), nor in comparison with Group 4 (Perceived Majority and Not Fused), except in community participation, where Group 1 scored lower than Group 3. Lastly, Group 3 (Perceived Majority and Fused) scored statistically significantly more than Group 2 (Perceived Minority and Not Fused) on all variables, excepting social contribution and social actualization.

In summary, the participants who perceive themselves as a linguistic minority in their surroundings and are not fused score less on the CPE, on well-being in general (except for social contribution and social actualization) and on community participation than participants who are fused and perceive themselves as a linguistic majority. However, scores on CPE, personal well-being and social integration of participants who also perceive themselves as a linguistic minority but who are fused, are comparable to the scores of participants who perceive themselves as a linguistic majority.

## Discussion

Even though group identification has been acknowledged as one of the main driving forces behind empowerment (Gutiérrez, 1995; van Zomeren et al., 2008; Drury and Reicher, 2009), studies on the effects of ethnic identity and PE are still scarce (Hipolito-Delgado and Zion, 2017; Lardier, 2018; Lardier et al., 2018; Molix and Bettencourt, 2010; Tamanas, 2010). Moreover, to our knowledge, there are no prior studies that studied empowerment (or efficacy) from a purely collective perspective in relation to ethnic identity and its psychological effects. The objective of this study was to analyze, in a singular population (i.e., an ethnic group in a situation of linguistic minority but not stigmatized), the relations of the identification as Basque speakers (or EIF) and CPE with personal well-being, social well-being and community participation. The results showed that individuals who are highly identified or fused with Basque speakers and who are highly empowered had higher indices of well-being (both personal and social) and of community participation than non-fused individuals with low empowerment.

### Association of Ethnic Identity Fusion and Empowerment (or Efficacy) With Well-Being and Community Participation

As hypothesized, the association between EIF and CPE was significant. In general, its size was less than the association between ethnic identity and PE (individual) found in other studies (Tamanas, 2010; Lardier, 2018; Lardier et al., 2018, 2019), although the difference was only statistically significant in comparison with the study by Lardier et al. (2018). It should be noted that the samples in these studies were mainly formed by young African Americans, and the measurements used were specific to measure ethnic identity. It is possible that age (Smith and Silva, 2011; Rivas-Drake et al., 2014), a more vulnerable socioeconomic situation (Morrison et al., 2011) and/or the instruments used influenced the relation between ethnic identity and empowerment. Moreover, we should also take into account that the empowerment measured in these studies was analyzed in individual, and not collective, terms.

On the other hand, regarding the other correlations, it should be highlighted that EIF, as a continuous variable, showed positive correlations with effect sizes that fell under the ranges found in meta-analyses on personal well-being (Smith and Silva, 2011) and social well-being (Espinosa et al., 2015), as well as within the ranges found in certain studies on community participation (Tamanas, 2010; Speer et al., 2013; Christens and Lin, 2014; Lardier, 2018; Lardier et al., 2018). Therefore, it could be argued that the association between ethnic identification and the personal well-being of Basques in this study is comparable to that found in studies conducted with people of color in the United States, for example, African Americans, Asian Americans, and Hispanic (Smith and Silva, 2011). Furthermore, the associations of EIF as a categorical variable (fused-not fused) were always slightly lower (except for community participation), and in this study, there is no evidence that confirms what Swann et al. (2009) suggest regarding the existence of a qualitative difference between fused and not-fused individuals.

Collective psychological empowerment had a high correlation with personal well-being, as well as social well-being, and medium-low correlation with community participation. Social factors, such as social well-being tend to be related to personal well-being (Keyes, 2006), as occurs in this study. In any event, the fact that CPE shows such a high correlation with personal well-being in this study is worthy of reflection. On the one hand, given that PE and CPE may be related (Staples, 1990; Bandura, 1997; Roos et al., 2013; Soares et al., 2015), it is possible that CPE was reflecting the PE of each individuals, such that the collective and its members reinforce (Staples, 1990) the perception of control or mastery over the environment (Martínez-Zelaya G. et al., 2015). This explanation, and in light of this work’s approach, where we have defended the importance of studying PE in collective terms, is clearly insufficient. In fact, the studies by Roos et al. (2013) and Soares et al. (2015), perceived collective efficacy and collective empowerment have proven to play an important role, even greater than the role of self-efficacy and individual empowerment in satisfaction and quality of life. Thus, collective empowerment may have a positive and prominent influence on both social and personal well-being. It may be that in certain populations such as the Basque, the collective aspects (in this case, CPE), take on an especially important role, not only in other social variables, but also in individual variables such as personal well-being. We bear in mind that the participants in this study are individuals who are committed to *Euskera* (a central aspect of Basque identity) and that, additionally, they have a very high identification or identity fusion scores, i.e., a self where the personal and the social aspects are very interwoven (Gómez and Vázquez, 2015). Thus, a desirable and necessary objective is to analyze the effects of PE and CPE on personal and social well-being in a differentiated fashion, and to study all these variables in other populations with a lower degree of identification or identity fusion. We will discuss these objectives later on.

### Synergy or Interaction Between the Fusion of Ethnic Identity and Collective Psychological Empowerment

The results show that fused and highly empowered participants have higher indices of personal well-being, social well-being and community participation than the rest of participants. However, the data show that CPE holds greater weight than EIF over the criterion variables. In fact, the hypothesis of interaction or synergy is partially supported by the contrast and *post hoc* analyses (although not by the MANOVA), only for the social integration and community participation dimension. This may be due to the fact that the participants in this study are individuals who actively defend *Euskera*, with a high degree of identification with Basque speakers. As such, it makes sense that this identification is more related to social integration (feeling of belonging and being accepted), but not so much with other variables. In turn, just as the meta-analysis by Talò (2018) showed, the factors most associated with community participation are forms of social identification, such as sense of community and collective identity, among others. These results are in line with what was found in the studies by Lardier et al. (2018) and Molix and Bettencourt (2010), in which significant relationships were also found between identity, empowerment, well-being, and community participation for American ethnic minorities (mainly youth). However, it is interesting to note that these relationships did not exist among white Americans (Molix and Bettencourt, 2010). The authors suggest that this disparity may be due to the fact that the acceptance of group identity as positive may be a coping strategy to mitigate the negative effects of social stigma and promote well-being for ethnic minorities, whereas for white Americans this coping strategy would not serve, as they lack this social stigma. This explanation, however, does not serve to understand these associations in the Basque community, since this group not only enjoys a good socio-economic position, but has managed to turn the shame of knowing Basque into pride. Later on we will try to give a plausible explanation to understand why these relationships also occur in the Basque community.

### Minority-Majority Perception and EIF

*Post hoc* results suggest that, just as shown in the results, being fused with Basque speakers and perceiving that one’s social group is a majority in the immediate environment has a positive effect on well-being and on community participation, and that not being fused and perceiving that Basque speakers are a minority has a negative effect. In addition, another result that is worth highlighting for its own importance is that in participants who have a fused identity, the negative effect of perceiving themselves as a minority is mitigated in the following variables: in CPE, in personal well-being, in the social integration dimension, and in community participation, while when the environment is perceived as a Basque-speaking majority, it appears that the effect of identification is diluted. These results are in line with studies that maintain that ethnic identification can be an effective tool to confront the effects associated with being a minority, and that, as such, defend ethnic identification as a particularly important factor for minority and/or discriminated groups (Crocker et al., 1994; Phinney et al., 1996; Branscombe et al., 1999; Mossakowski, 2003; Carter et al., 2005; Molix and Bettencourt, 2010).

In order to understand what these results mean, it is necessary to take into account that the social groups usually studied in this area, including empowerment tend to be vulnerable collectives (Gutiérrez, 1995; Karaś et al., 2015; Lardier, 2018; Lardier et al., 2018), while the Basque collective possesses a series of particularities that should be considered separately. The achievements in terms of culture, and especially language, are clearly coherent with the Basque collective’s perception of power to change society and reach its objectives. However, these achievements, as well as the end of the Basque conflict, are very recent and, therefore, we still find certain indicators of vulnerability, such as the fragile situation of *Euskera* (Sociolinguistic Cluster, 2020) and specific indicators of discrimination against the language in some contexts (Gorter et al., 2012). Furthermore, at present, the political actions of the Spanish and French governments are sometimes perceived as a threat to the survival of *Euskera*, and therefore, to the Basque identity.

According to the social identity theory, when a collective is a minority and perceives relevant threats that concern its identity, the identity is activated (Crocker and Luhtanen, 1990) and this can begin empowerment processes (Gutiérrez, 1995). It may be that, despite the generally good socioeconomic position of this social group and the achievements reached as far as the language is concerned, the awareness of being a linguistic minority both in the world and within the population of the Basque Country as a whole, contributes to the fact that the identification or degree of identity fusion with Basque speakers and, especially, empowerment, act as especially important factors for well-being.

### Limitations and Future Research

This study has a series of limitations that should be addressed. Firstly, this is a cross-sectional study, so it is not possible to establish a temporal relation between the variables with certainty. Furthermore, this work has other limitations that should be considered in future research.

On the one hand, it is a convenience sample and cannot be considered a representative sample of the Basque population. Furthermore, the sample is not sufficiently varied in terms of EIF. The average identity fusion of participants is very high, 4.28 (*SD* = 0.77, on a scale of 1 to 5). Participants in this study are active individuals who are in the habit of running in *Korrika*, which, as already mentioned, is a mass recreational race to defend and promote the Basque language. As such, it is very likely that this sample does not represent the variability in EIF that we might find in the general Basque population. Also, the sample was composed mostly by women. This may be due to the fact that women are usually the main transmitters and promoters of culture and language, and in this specific case of Korrika and Euskera (Del Valle, 1988). Additionally, although the age of participants in this study reflected great variability (ranging between 18 and 73 years), not all age ranges were equally represented. In the future, it would be desirable to study these same relations in a sample of Basque citizens with higher variability in EIF, as well as in a more balanced sample regarding gender and age.

Regarding the scale used to measure EPC, in future studies, it would be advisable to also consider including elements that capture the affect associated with the perception of the capacity to enhance change and greater social justice (Drury and Reicher, 2009), as well as measures for sociopolitical control (Peterson et al., 2011), that provide a better measurement of empowerment in collective terms. Additionally, it would be enriching to consider including both types of empowerment, individual and collective, and to analyze the relation between them, as well as their effects.

Another issue of interest, given the aforementioned particularities of the population of this study, is the possible effect of the perception of a threat to, or discrimination against this population. Although a variable related to linguistic minority-majority perception was included, it would also be desirable to include measures designed to more directly assess the perception of being a discriminated or disadvantaged group, since this perception could be a key factor to clarify the relations between EIF, CPE and well-being (Crocker et al., 1994; Phinney et al., 1996; Branscombe et al., 1999; Mossakowski, 2003; Carter et al., 2005; Molix and Bettencourt, 2010).

Finally, regarding future research, it is important to consider other factors analyzed in previous studies and that may influence relations between social identification and well-being: i) size and nature of the group targeted in the study, including proximal groups (organizational groups, volunteer groups, friends, family, community, town, place, etc.) or larger and distal (nation, ethnicity, race, gender, religion, etc.) (Postmes et al., 2019); ii) socioeconomic status of the individuals themselves; iii) the political, socioeconomic (per capita income, human development index, distance from power, etc.) and cultural (individualism-collectivism) characteristics of the country and the group targeted by the study (Morrison et al., 2011; Espinosa et al., 2015).

## Conclusion

Based on the results of this work, it is possible to conclude that CPE, along with social identification (or identity fusion) are important factors that positively influence well-being (both personal and social) and community participation, with empowerment standing out as especially noteworthy. On one hand, considering the role of empowerment in this study, and on the other, this collective’s characteristics (which are unusual in this field), we believe that this work contributes to the emerging knowledge about the relation between social identification (or identity fusion) and empowerment and its influence on well-being and community participation.

## Data Availability Statement

The original contributions presented in the study are included in the article/supplementary material, further inquiries can be directed to the corresponding author.

## Ethics Statement

The studies involving human participants were reviewed and approved by University of the Basque Country’s Ethics Committee for Research Involving Human Beings. The patients/participants provided their written informed consent to participate in this study.

## Author Contributions

JZ, SC, and AP contributed to the design and implementation of the research, and analyzed and interpreted the data. JZ collected the study’s data. All the authors discussed the results, and contributed to the writing and editing of the manuscript.

## Conflict of Interest

The authors declare that the research was conducted in the absence of any commercial or financial relationships that could be construed as a potential conflict of interest.

## Footnotes

1 Ethnic identity is “a complex construct including a commitment and sense of belonging to the group, positive evaluation of the group, interest in and knowledge about the group, and involvement in social activities of the group” (Phinney et al., 1996, p. 168).

2 Euskera is an isolated language whose roots bear no relation to Indo-European languages. Its origin, as well as the origin of the Basques themselves, is a riddle whose solution continues to challenge many scholars.

3 For example, Americans of European origin may be considered as members of (majority) ethnic groups (Phinney, 1996).
